# Niacinamide and undenatured type II collagen modulates the inflammatory response in rats with monoiodoacetate-induced osteoarthritis

**DOI:** 10.1038/s41598-021-94142-3

**Published:** 2021-07-19

**Authors:** Kazim Sahin, Osman Kucuk, Cemal Orhan, Mehmet Tuzcu, Ali Said Durmus, Ibrahim Hanifi Ozercan, Nurhan Sahin, Vijaya Juturu

**Affiliations:** 1 Department of Animal Nutrition, Faculty of Veterinary Medicine, Firat University, Elazig, Turkey; 2 Department of Animal Nutrition, Faculty of Veterinary Medicine, Erciyes University, Kayseri, Turkey; 3 Department of Biology, Faculty of Science, Firat University, Elazig, Turkey; 4 Department of Surgery, Faculty of Veterinary Medicine, Firat University, Elazig, Turkey; 5 Department of Pathology, Faculty of Medicine, Firat University, Elazig, Turkey; 6 Lonza Inc., Consumer Health and Nutrition, Morristown, NJ USA

**Keywords:** Biochemistry, Immunology, Medical research

## Abstract

The current work aimed to examine the properties of oral supplementation of niacinamide and undenatured type II collagen (UCII) on the inflammation and joint pain behavior of rats with osteoarthritis (OA). Forty-nine Wistar rats were allocated into seven groups; control (no MIA), MIA as a non-supplemental group with monosodium iodoacetate (MIA)-induced knee osteoarthritis, MIA + undenatured type II collagen (UCII) at 4 mg/kg BW, MIA + Niacinamide at 40 mg/kg BW (NA40), MIA + Niacinamide at 200 mg/kg BW (NA200), MIA + UCII + NA40 and MIA + UCII + NA200. Serum IL‐1β, IL‐6, TNF-α, COMP, and CRP increased in rats with OA and decreased in UCII and NA groups (*p* < 0.05). Rats with osteoarthritis had greater serum MDA and knee joint MMP-3, NF-κB, and TGβ protein levels and decreased in treated groups with UCII and NA (*p* < 0.05). The rats with OA also bore elevated joint diameters with joint pain behavior measured as decreased the stride lengths, the paw areas, and the paw widths, and increased the Kellgren-Lawrence and the Mankin scores (*p* < 0.05) and decreased in UCII treated groups. These results suggest the combinations with the UCII + NA supplementation as being most effective and reduce the inflammation responses for most OA symptoms in rats.

## Introduction

Osteoarthritis (OA) is a degenerative condition of articular cartilage, in which the knee is the most affected joint. Over 10% of the world population suffers from knee OA, including 14 million Americans^1^. The patients with knee OA go through surgical treatment (arthroscopy and total knee arthroplasty) due to the pain, stiffness, and deformation typically seen in the disease^2^. However, up to 20% of patients complain of persisting pain after surgery^3^. Therefore, preventive thoughts, including relevant nutrient supplementations to support the joints, should also be considered for OA of all kinds.

Niacinamide is the amide form of vitamin B3 (niacin) not only involves in the synthesis of NAD+, repairmen of damaged DNA and pigmentary disorders, and being part of antioxidant defense mechanisms^4–6^, but also takes part in the regulation of cellular inflammation, which leads to arthritis through the inhibition of collagen II expression^7^. In addition, Jonas et al.^8^ found that niacinamide held a beneficial role in treating osteoarthritis measured as better joint flexibility and decreased inflammation and arthritis impact (arthritis severity).

As the main part of collagen fibrils in hyaline cartilage of the articular surfaces, type II collagen is also present in the nucleus pulposo of the intervertebral disc and vitreous of the eye. Natural type II collagen derived from chicken sternum cartilage has been revealed to be beneficial in patients with rheumatoid arthritis^9,10^ as well as patients with knee osteoarthritis^11^. Undenatured type II collagen (UCII) is also a native type II collagen derived from chicken sternum cartilage^12^ and has been proven to improve OA symptoms in dogs^13^.

Niacinamide prevents cytokine-mediated induction of nitric oxide synthase, thus, decreases inflammation in various cell types^14^. Knockout of the GPR109a gene encoding the niacin receptor led to a reduction in Foxp3 + T cells (regulatory T cells or Tregs), increases of CD4 + T cells producing IL-10 and IL-18, increases of CD4 + T cells producing proinflammatory cytokine IL-17, and the inability of CD 103 + to induce the Treg differentiation in vitro^15^. These effects suggest that niacin may influence the Treg activation leading to restoration of the joint deterioration. Therefore, the niacin receptor knockout led to the downregulation of the T regulator pathway modulated by UCII, potential synergy/additivity. Although this receptor (GPR109a) may have a high affinity for niacin^16^, this can possibly be a shared pathway between both UCII and niacinamide and may show some synergy between both ingredients. In addition, nicotinates have been described to inhibit the SIRT1, a biomarker for joint also linked to UCII^17^. Niacin could affect the oral tolerance pathway similarly to UCII. A combination of UCII and niacin may be used as a joint health product to lead to better efficacy (additivity/synergy).

The number of works conducted on the effects of niacinamide and UCII as single supplementations is just a few in the literature, and the combination of the two supplements has not been investigated in humans or animal models for knee OA. The rationale for the present work was a need for an alternative as a combination of collagen and niacinamide in the treatment of OA in terms of relieving the pain and/or other symptoms. Therefore, the objective of this work was to examine the properties of niacinamide and UCII supplementation as single or as a combination on some serum biochemical and inflammation parameters, MDA and antioxidant enzymes levels, and stride lengths, paw areas, diameters, and inflammation parameters of the knee joint along with histopathologic and radiographic images in monosodium iodoacetate (MIA)-induced knee osteoarthritis of rat models**.**

## Materials and methods

### Animals and experimental design

Male Wistar rats with eight weeks (mean weight of 180 ± 200 g) were purchased from Firat University Experimental Research Centre. Animals were housed in cages of three to five rats with a 12 light-12 h dark cycle at constant temperature and humidity. The research was approved by the Animal Ethics Committee of Firat University (2019/88-135) and all experimental methods were conducted in accordance with relavent ethical guidelines for laboratory animal use and care^18^. The present study was also carried out in compliance with the ARRIVE guidelines. All animals were given ad libitum to feed and water.

Forty-nine male Wistar albino rats were randomly allocated into seven groups (n = 7 each), namely; Control as a non-supplemental group with no osteoarthritis-induced rats, MIA as a non-supplemental group with 1 mg monosodium iodoacetate (MIA)-induced knee osteoarthritis, MIA + UCII as MIA group gavage-fed a supplemental undenatured type II collagen (UCII) at 4 mg/kg BW, MIA + NA40 as MIA group gavage-fed supplemental niacinamide (NA) at 40 mg/kg, MIA + NA200 as MIA group gavage-fed a supplemental NA at 200 mg/kg, MIA + UCII + NA40 as MIA group gavage-fed both supplemental UCII at 4 mg/kg mg/kg and NA at 40 mg/kg and MIA + UCII + NA200 as MIA group gavage-fed both supplemental UCII at 4 mg/kg mg/kg and NA at 200 mg/kg.

The OA rat model was performed as previously described^19,20^. To induce OA rat model, the right knee of the rats was shaved and disinfected with 70% alcohol following anaesthetization using xylazine (10 mg/kg) and ketamine hydrochloride (50 mg/kg). 1.0 mg of MIA (Sigma, St. Louis, U.S.A.) was dissolved in 50 μL saline and injected into right knee joints through the infrapatellar ligament using a 0.3 ml insulin syringe fitted with a 29-G needle. The control group received an injection of 50 μL saline. A week before injection with MIA, the niacinamide at 40 or 200 mg/kg BW and UCII (Lonza, New Jersey, U.S.A.) at 4 mg/kg BW were delivered through oral gavage until day 30 (i.e., from d7 to d30). The regular diet and water were offered ad libitum. The dose of niacinamide use at the present work was determined based on the work published in the literature^21,22^, and the dose of 4 mg UCII was calculated based on a previous study^23^.

### Measurement of joint swelling (edema)

All rats were observed every other alternate day to assess knee joint swelling. The clinical assessment consisted of pain evaluation and inflammation by measuring joint diameter size. Three right knee joint thickness measures were taken under anesthesia using an electronic digital caliper. The results were expressed as an average in mm.

### Gait test

Gait test (paw area, paw width, stride length) of the knee joint was analyzed. The ink was smeared on the hind paws, and rats were permitted to run on a 60 cm long and 7 cm wide path covered with white paper. A dark chamber was located at the end of the road to persuade the animals. Upon the end of the test, the paper was scanned at 300dpi. The size around the paw was described as paw area (cm^2^), the distance between the first and fifth toes as paw width (cm), the distance of the same hind paw between two steps as stride length (cm). The footsteps were measured by Image J software (version 1.43u, National Institutes of Health, USA).

### Determination of the Kellgren–Lawrence score and cartilage evaluation

Experienced senior radiologists determined the severity of OA in all rats. The severity in each joint was evaluated according to the Kellgren–Lawrence scoring system^24^ (Table 1). The extent of articular cartilage damage for each joint compartment was assessed using the Mankin system^25^ by an experienced senior surgeon who was blind to the study groups (Table 2).

**Table 1:** Kellgren–Lawrence scoring system^24^.

Stage	Radiologic findings
0	None
1	Doubtful: Suspicious narrowing of the joint space and possible osteophyte formation
2	Minimal: Definite osteophyte and possible narrowing of the joint space
3	Moderate: Numerous moderate osteophytes, definite narrowing of the joint space, some sclerosis, and possible deformity of the bone ends
4	Severe: Large osteophytes, marked narrowing of the joint space, sclerosis, and deformity of the bone ends

**Table 2:** Cartilage evaluation according to the Mankin system^25^.

Criteria	Score	Histological finding
Structure	0	Smooth intact surface
	1	Slight surface irregularities
	2	Pannus/surface fibrillation
	3	Clefts into the transitional zone
	4	Clefts into the radial zone
	5	Clefts into the calcified zone
	6	Total disorganization
Cells	0	Uniform cell distribution
	1	Diffuse cell proliferation
	2	Cell clustering
	3	Cell loss
Tidemark integrity	0	Intact
	1	Vascularity

### Biochemical analysis

At the end of the study, the rats were sacrificed, and blood samples were collected. The blood samples were centrifuged, and the collected sera were kept at − 80 °C. Serum biochemical parameters, namely glucose, blood urea nitrogen (BUN), and creatine levels, as well as ALT and AST activities, were assessed biochemistry analyzer (Samsung Electronics Co., Suwon, Korea). Enzyme-linked immunosorbent assay (ELISA) kits (Cayman Chemical, Ann Arbor, MI, USA) were used in analyzing serum inflammation parameters of IL‐1β, IL‐6, TNF-α, cartilage oligomeric matrix protein (COMP), and C-reactive protein (CRP) according to the manufacturer instructions. Serum malondialdehyde (MDA) was analyzed using an HPLC apparatus of Shimadzu (Shimadzu, Japan) equipped with UV–vis SPD-10 AVP detector, a CTO-10 AS VP column, and 30 mM KH_2_PO_4_ and methanol (82.5: 17.5, v/v, pH 3.6) at a flow rate of 1.2 mL/min^26^. Column waste was monitored at 250 nm. Antioxidant levels of superoxide dismutase (SOD), catalase (CAT), and glutathione peroxidase (GPx) were measured using the relevant commercial kits (Cayman Chemical, Ann Arbor, MI, USA) according to the ELISA method.

### Western blot analysis

Joint tissue protein levels (IL-1β, IL-6, IL-10, TNF-α, COMP, collagen type II, MMP-3, NF-κB, and TGF)-β1 levels from the articular cartilage samples were analyzed using the Western blot technique as defined by Yabas et al.^27^. Firstly, joint tissue samples were homogenized and 20 μg of protein was electrophoresed and transferred to a nitrocellulose membrane. The membranes were incubated with primary antibodies (IL-1β, IL-6, IL-10, TNF-α, COMP, MMP-3, and NFkB; Abcam, Cambridge, UK) that were diluted. In the following stage, nitrocellulose membranes were incubated with a peroxidase-conjugated secondary antibody. Finally, the relative densities of the bands, visualized by diaminobenzidine solution, were examined using the Image analysis system (Image J National Institute of Health Bethesda, USA). Data are expressed as a percent of the control. Full blots are included in the supplementary file (Supplementary Fig. S2,S3).

### Histological evaluates

Histological alterations were assessed to check the effects of the product on cartilage degeneration in the knee joints of MIA-induced OA rats. Following the rat sacrifice, each knee joint was resected, fixed in 10% formalin for 24 h at 4 °C, and decalcified with 5% hydrochloric acid for four days at 4 °C. Following decalcification, specimens were dehydrated in graded acetone and embedded in paraffin. Sections (thickness, 2–3 µm) were stained with 0.2% hematoxylin and 1% eosin (H&E) for 5 min and 3 min, respectively. The histological preparations were analyzed and photographed with a microscope using a digital image capture camera by an experienced histopathologist blind to the study groups.

### Statistical analyses

The sample size of the work was figured out by the G* Power program (Version 3.1.9.2) with alpha error 0.05 and 85% power with effect size 0.65 calculated from earlier studies^28,29^. In this study, conformism to normality from the prerequisites of the parametric tests was implemented using the “Shapiro–Wilk” test, and the homogeneity of the variances was checked with the “Levene” test. Analysis of variance (ANOVA) test was performed to determine the differences between the groups, and post-hoc Tukey test was used for multiple comparisons of the groups. For nonparametric data, the radiologic and histopathologic scores were analyzed using Kruskal–Wallis followed by Mann–Whitney U. Statistical significance was accepted as *p* < 0.05.

### Ethics approval and consent to participate

The research was approved by the Animal Ethics Committee of Firat University (2019/88-135) and conducted following the ethical guidelines for laboratory animal use and care.

## Results

Serum glucose, BUN, and creatine levels as well as ALT and AST activities, remained unchanged among treatments (*p* > 0.05; Table 3). Serum IL‐1β, IL‐6, TNF-α, COMP, and CRP concentrations increased in rats with OA compared with control rats (*p* < 0.05; Table 4). Supplementing UCII, NA40, and NA200 alone equally reduced the measured concentrations (*p* < 0.05). However, the combination of UCII and NA at both 40 and 200 mg/kg treatments equally provided further decreases in the concentrations of the inflammation parameters. Numerically, the UCII + NA40 treatment provided the lowest IL‐1β concentrations compared with that of MIA.

**Table 3:** Effects of niacinamide (NA) and undenatured type II collagen (UCII) on serum biochemical parameters in rats (n = 7).

Items	Groups						
	Control	MIA	MIA + UCII	MIA + NA40	MIA + NA200	MIA + UCII + NA40	MIA + UCII + NA200
Glucose (mg/dL)	114.71 ± 5.31	116.57 ± 7.28	116.00 ± 10.71	114.71 ± 7.48	116.14 ± 8.11	115.14 ± 9.67	116.43 ± 4.31
BUN (mg/dL)	24.24 ± 3.02	24.24 ± 0.71	23.10 ± 4.20	24.37 ± 2.20	24.30 ± 2.28	24.63 ± 0.78	24.10 ± 2.71
Creatine (mg/dL)	0.48 ± 0.10	0.47 ± 0.10	0.48 ± 0.10	0.48 ± 0.09	0.46 ± 0.08	0.46 ± 0.11	0.47 ± 0.11
ALT (U/L)	70.57 ± 8.30	68.29 ± 4.72	70.29 ± 5.88	69.86 ± 3.89	71.71 ± 6.97	72.14 ± 11.78	69.00 ± 3.70
AST (U/L)	88.43 ± 12.99	87.00 ± 11.85	87.43 ± 12.47	88.14 ± 11.17	86.86 ± 6.47	89.57 ± 6.50	85.14 ± 6.96

Data are presented as mean and standard deviation (*p* > 0.05; ANOVA and Tukey's post-hoc test). NA: niacinamide; MIA: monosodium iodoacetate; UCII: undenatured type II collagen; BUN: Blood urea nitrogen; ALT: Alanine aminotransferase; AST: Aspartate aminotransferase. NA40 and NA200 represent 40 and 200 mg/kg niacinamide dose applications, respectively.

**Table 4:** Effects of niacinamide (NA) and undenatured type II collagen (UCII) supplementation on serum inflammation parameters in rats (n = 7).

Items	Groups						
	Control	MIA	MIA + UCII	MIA + NA40	MIA + NA200	MIA + UCII + NA40	MIA + UCII + NA200
IL-1β (pg/mL)	20.13 ± 2.86^d^	47.62 ± 4.63^a^	36.59 ± 2.43^b^	38.78 ± 3.36^b^	35.82 ± 2.04^b^	24.97 ± 3.65^cd^	26.02 ± 3.86^c^
IL-6 (pg/mL)	8.92 ± 1.47^d^	39.46 ± 1.83^a^	31.00 ± 1.85^b^	32.72 ± 2.98^b^	31.47 ± 2.72^b^	21.18 ± 2.38^c^	20.23 ± 1.99^c^
TNF-α (pg/mL)	24.02 ± 3.60^d^	68.03 ± 3.41^a^	47.17 ± 3.15^b^	51.10 ± 5.67^b^	49.06 ± 3.76^b^	34.27 ± 6.46^c^	31.62 ± 2.21^c^
COMP (pg/mL)	7.28 ± 1.06^d^	32.10 ± 3.03^a^	25.76 ± 2.98^b^	27.69 ± 2.31^b^	26.40 ± 3.07^b^	16.72 ± 1.71^c^	15.10 ± 2.46^c^
CRP (pg/mL)	1.79 ± 0.19^d^	10.76 ± 1.45^a^	7.18 ± 0.67^b^	7.54 ± 0.61^b^	7.17 ± 0.64^b^	4.39 ± 0.58^c^	4.23 ± 0.64^c^

Data are presented as mean and standard deviation (p > 0.05; ANOVA and Tukey's post-hoc test). NA: niacinamide; MIA: monosodium iodoacetate; UCII: undenatured type II collagen; IL-1β, Interleukin 1 beta; IL-6, Interleukin 6; TNF-α, tumor necrosis factor-alpha; COMP, cartilage oligomeric matrix protein; CRP, C-reactive protein. NA40 and NA200 represent 40 and 200 mg/kg niacinamide dose applications, respectively. (a–d): Means in the same line without a common superscript differ significantly.

Rats with osteoarthritis had greater MDA but lower SOD, CAT, and GPx activities compared with those of rats in the control group (*p* < 0.05; Table 5). However, the rats received each supplementation as single or as a combination except for UCII treatments, which were similar to those of MIA, which reversed the responses (*p* < 0.05). Numerically but not statistically, MDA concentrations were lowest with the treatment of NA200, although concentrations of SOD, CAT, and GPx were greatest with UCII + NA200 treatment compared with those of MIA.

**Table 5:** Effects of niacinamide (NA) and undenatured type II collagen (UCII) supplementation on serum MDA and antioxidant enzymes levels in rats (n = 7).

Items	Groups						
	Control	MIA	MIA + UCII	MIA + NA40	MIA + NA200	MIA + UCII + NA40	MIA + UCII + NA200
MDA (nmol/mL)	0.71 ± 0.08^d^	2.94 ± 0.11^a^	2.81 ± 0.12^a^	2.28 ± 0.11^b^	2.00 ± 0.08^c^	2.35 ± 0.13^b^	2.31 ± 0.31^b^
SOD (U/mL)	81.2 ± 5.08^a^	39.05 ± 4.53^d^	42.19 ± 3.27^cd^	50.20 ± 7.90^bc^	53.24 ± 4.62^b^	52.29 ± 6.85^b^	56.69 ± 4.06^b^
GPx (U/mL)	58.97 ± 2.80^a^	20.01 ± 2.01^d^	22.41 ± 2.41^d^	27.84 ± 1.48^c^	30.10 ± 2.94^bc^	30.36 ± 2.36^bc^	32.68 ± 2.54^b^
CAT (U/mL)	159.29 ± 7.90^a^	110.18 ± 7.95^c^	113.18 ± 5.37^c^	122.48 ± 3.93^b^	128.88 ± 3.52^b^	129.34 ± 3.96^b^	130.93 ± 3.94^b^

Data are presented as mean and standard deviation (*p* > 0.05; ANOVA and Tukey's post-hoc test). NA: niacinamide; MIA: monosodium iodoacetate; UCII: undenatured type II collagen; MDA, malondialdehyde; SOD, superoxide dismutase; CAT, catalase; GPx, glutathione peroxidase. NA40 and NA200 represent 40 and 200 mg/kg niacinamide dose applications, respectively. (a–d): Means in the same line without a common superscript differ significantly.

**Figure 1:**
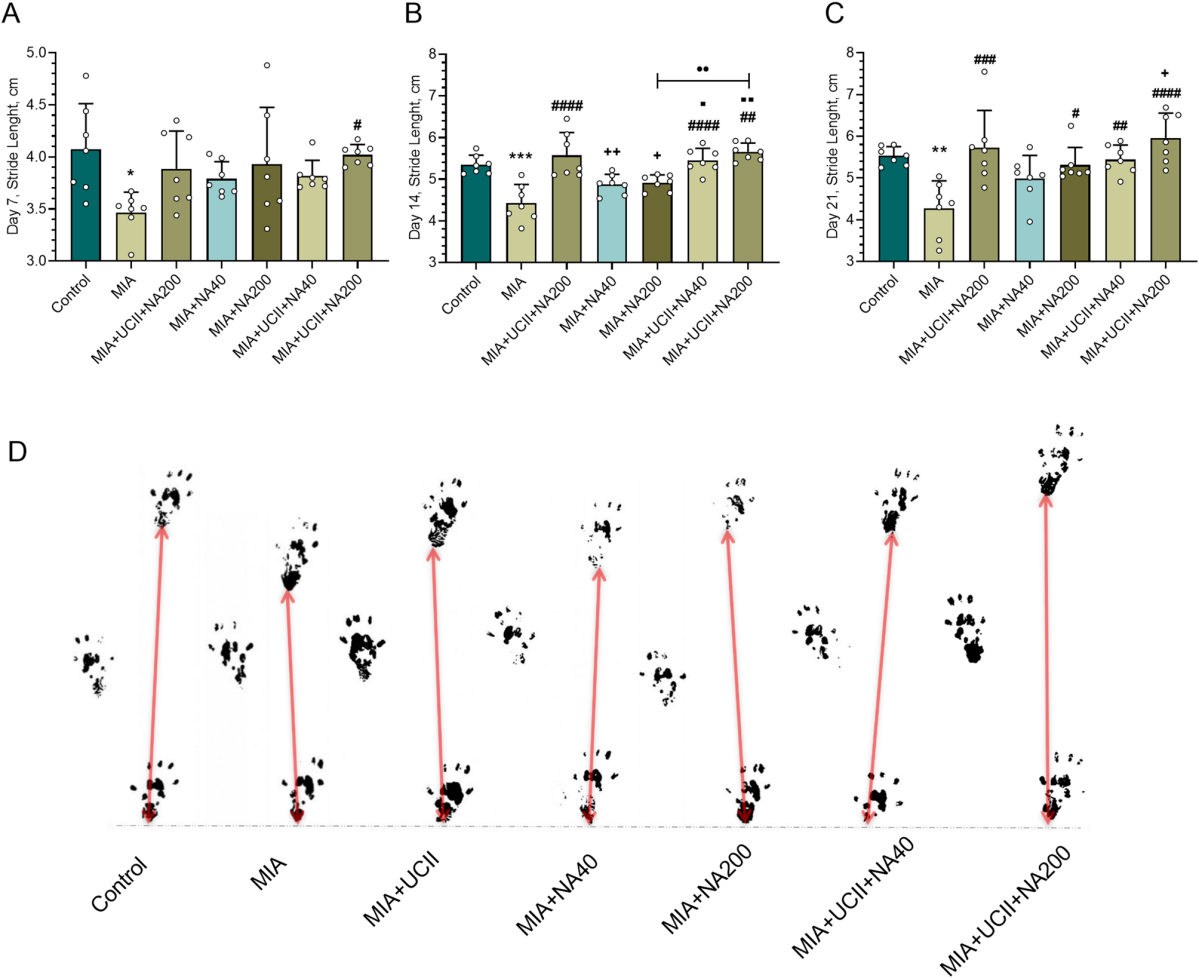
Effects of niacinamide (NA) and undenatured type II collagen (UCII) supplementation on stride length [on days 7 (**A**), 14 (**B**), and 21 (**C**)] in monosodium iodoacetate (MIA)-induced osteoarthritis in rats (n = 7). Representative images of the stride length measured on day 21 of the study are shown (**D**). Control as a non-supplemental group with no osteoarthritis-induced rats, MIA as a non-supplemental group with monosodium iodoacetate (MIA)-induced knee osteoarthritis, MIA + UCII as MIA group gavage-fed a supplemental UCII at 4 mg/kg, MIA + NA40 as MIA group gavage-fed supplemental niacinamide (NA) at 40 mg/kg, MIA + NA200 as MIA group gavage-fed a supplemental NA at 200 mg/kg, MIA + UCII + NA40 as MIA group gavage-fed both supplemental UCII at 4 mg/kg and NA at 40 mg/kg and MIA + UCII + NA200 as MIA group gavage-fed both supplemental UCII at 4 mg/kg and NA at 200 mg/kg. Oral gavage delivery of supplements was applied from d7 to d30. The error bars point out the standard deviation of the mean. ANOVA and Tukey’s post-hoc test were used to compare the results among different treatment groups. Statistical significance between groups is shown by: *p < 0.05; **p < 0.01; ***p < 0.001 compared as Control group and, ^#^*p* < 0.05; ^##^*p* < 0.01; ^###^*p* < 0.001; _####_*p* < 0.0001 compared as MIA group and, ^+^*p* < 0.05; ^++^*p* < 0.01 compared as MIA + UCII group and ^⬛^*p* < 0.05; ^⬛⬛^*p* < 0.01 compared as MIA + NA40 group and, ••*p* < 0.01 compared as pairwise comparisons between the groups) (ANOVA and Tukey's post-hoc test; *p* < 0.05).

The stride lengths and their representative images at d7, d14, and d21 are shown in Fig. 1. The stride length decreased in rats with OA compared to rats of control (*p* < 0.05) at d7, d14, and d21. The rats receiving supplements of UCII or NA increased the stride length with various degrees (*p* < 0.05) in comparison to those of MIA rats (*p* < 0.05), the treatment of the combination of UCII + NA200 providing the greatest stride length even greater than that of control at d14 and d21. The representative images from the rats of control or the treatments showed similar trends to those of stride lengths at d21.

**Figure 2:**
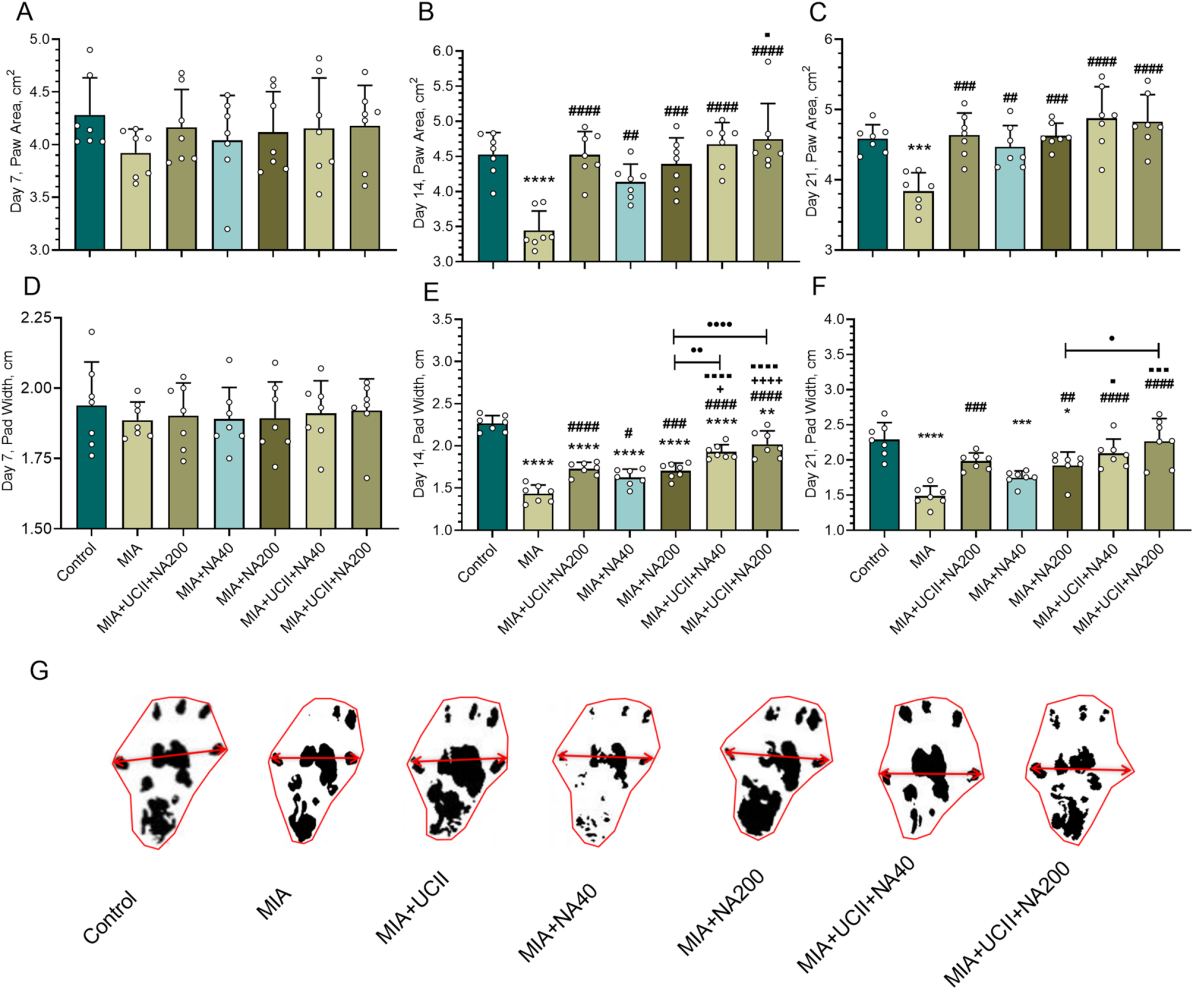
Effects of niacinamide (NA) and undenatured type II collagen (UCII) supplementation on paw area [on days 7 (**A**), 14 (**B**), and 21 (**C**)] and pad width [on days 7 (**D**), 14 (**E**), and 21 (**F**)] in monosodium iodoacetate (MIA)-induced osteoarthritis in rats (n = 7). Representative images of the paw area and pad width measured on day 21 of the study are shown (**G**). Control as a non-supplemental group with no osteoarthritis-induced rats, MIA as a non-supplemental group with monosodium iodoacetate (MIA)-induced knee osteoarthritis, MIA + UCII as MIA group gavage-fed a supplemental UCII at 4 mg/kg, MIA + NA40 as MIA group gavage-fed supplemental niacinamide (NA) at 40 mg/kg, MIA + NA200 as MIA group gavage-fed a supplemental NA at 200 mg/kg, MIA + UCII + NA40 as MIA group gavage-fed both supplemental UCII at 4 mg/kg and NA at 40 mg/kg, and MIA + UCII + NA200 as MIA group gavage-fed both supplemental UCII at 4 mg/kg and NA at 200 mg/kg. Oral gavage delivery of supplements was applied from d7 to d30. ANOVA and Tukey’s post-hoc test were used for comparing the results among different treatment groups. Statistical significance between groups is shown by: *p < 0.05; **p < 0.01; ***p < 0.001; ****p < 0.0001 compared as Control group and, ^#^*p* < 0.05; ^##^*p* < 0.01; ^###^*p* < 0.001; ^####^*p* < 0.0001 compared as MIA group and, ^+^*p* < 0.05; ^++++^*p* < 0.0001 compared as MIA + UCII group and, ^⬛^*p* < 0.05; ^⬛⬛⬛^*p* < 0.001; ^⬛⬛⬛⬛^*p* < 0.0001 compared as MIA + NA40 group and, •*p* < 0.05; ••*p* < 0.01; ••••*p* < 0.0001 compared as pairwise comparisons between the groups). (ANOVA and Tukey's post-hoc test; *p* < 0.05).

Although the paw areas and the paw widths remained similar among treatments at d7 (*p* > 0.05; Fig. 2), the rats with OA had reduced paw areas and paw widths at d14 and d21 (*p* < 0.05). The rats supplemented only with a combination of UCII + NA200 increased (*p* < 0.05) the paw areas at d14. However, all supplements equally increased the paw areas in rats with OA bringing the values to those of control at d21. The paw widths in rats with OA increased equally (*p* < 0.05) with single supplements, and further increases (*p* < 0.05) were equally observed with the combination of UCII and NA treatments at d14. Similar responses were also observed at d21, with the combination of UCII^+^NA200 treatment having the greatest paw width values even similar to those of control. The representative images of the paw area and pad width from the rats of control or the treatments showed similar trends to those of stride lengths at d21.

**Figure 3:**
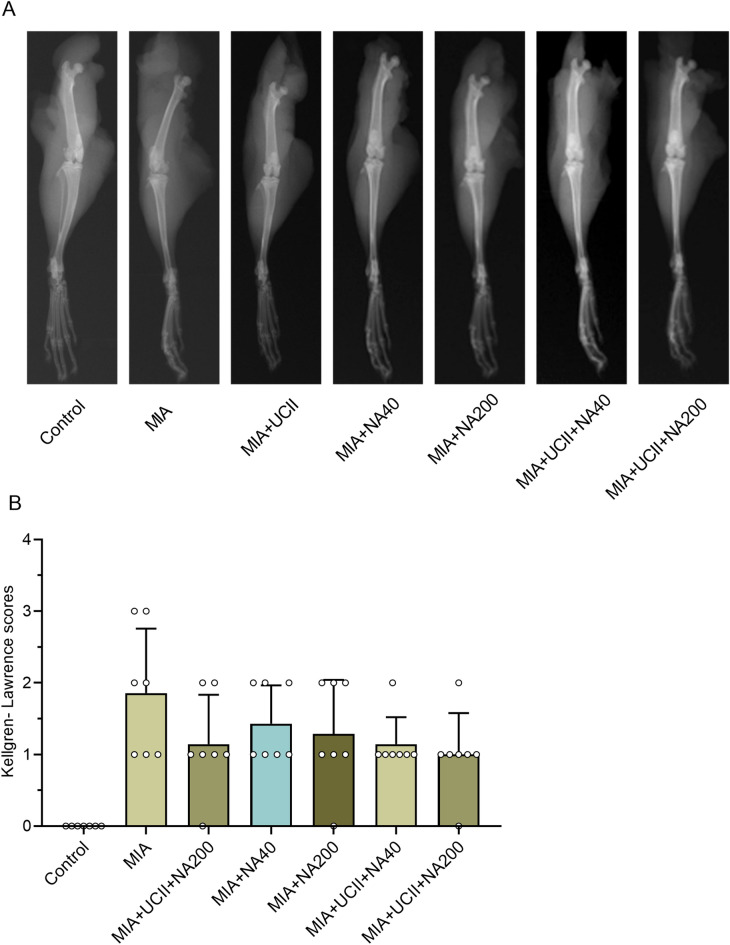
Effects of niacinamide (NA) and undenatured type II collagen (UCII) supplementation on knee joint in monosodium iodoacetate (MIA)-induced osteoarthritis in rats (n = 7). Representative radiographic images (A) obtained at the end of the experiment are shown. Mean values of Kellgren- Lawrence scores are demonstrated (B) with ± standard deviations (Kruskal–Wallis followed by Mann–Whitney U; *p* > 0.05). Control as a non-supplemental group with no osteoarthritis-induced rats, MIA as a non-supplemental group with monosodium iodoacetate (MIA)-induced knee osteoarthritis, MIA + UCII as MIA group gavage-fed a supplemental UCII at 4 mg/kg, MIA + NA40 as MIA group gavage-fed supplemental niacinamide (NA) at 40 mg/kg, MIA + NA200 as MIA group gavage-fed a supplemental NA at 200 mg/kg, MIA + UCII + NA40 as MIA group gavage-fed both supplemental UCII at 4 mg/kg and NA at 40 mg/kg and MIA + UCII + NA200 as MIA group gavage-fed both supplemental UCII at 4 mg/kg and NA at 200 mg/kg. Oral gavage delivery of supplements was applied from d7 to d30.

Representative radiographic images indicated minimal Kellgren-Lawrence scores (grade 0; Fig. 3) with preservation of the joint space along with no signs of joint space narrowing and no formation of osteophytes in intact rats. However, rats with OA had a high score reaching grade 3 with common radiographic features, including joint space, narrowing, reduced articular space, sclerosis with articular surface irregularity, and the intense formation of osteophytes and intense osteophyte formation. The rats treated with UCII and NA alone experienced less evidence of the common radiographic features of OA, along with the scores not exceeded grade 2. However, the rats treated with UCII + NA200 had further alleviation of the scores (grade 1) along with a normal thickness of the cartilage surfaces, being able to reduce the degree of knee joint involvement in relation substantially.

**Figure 4:**
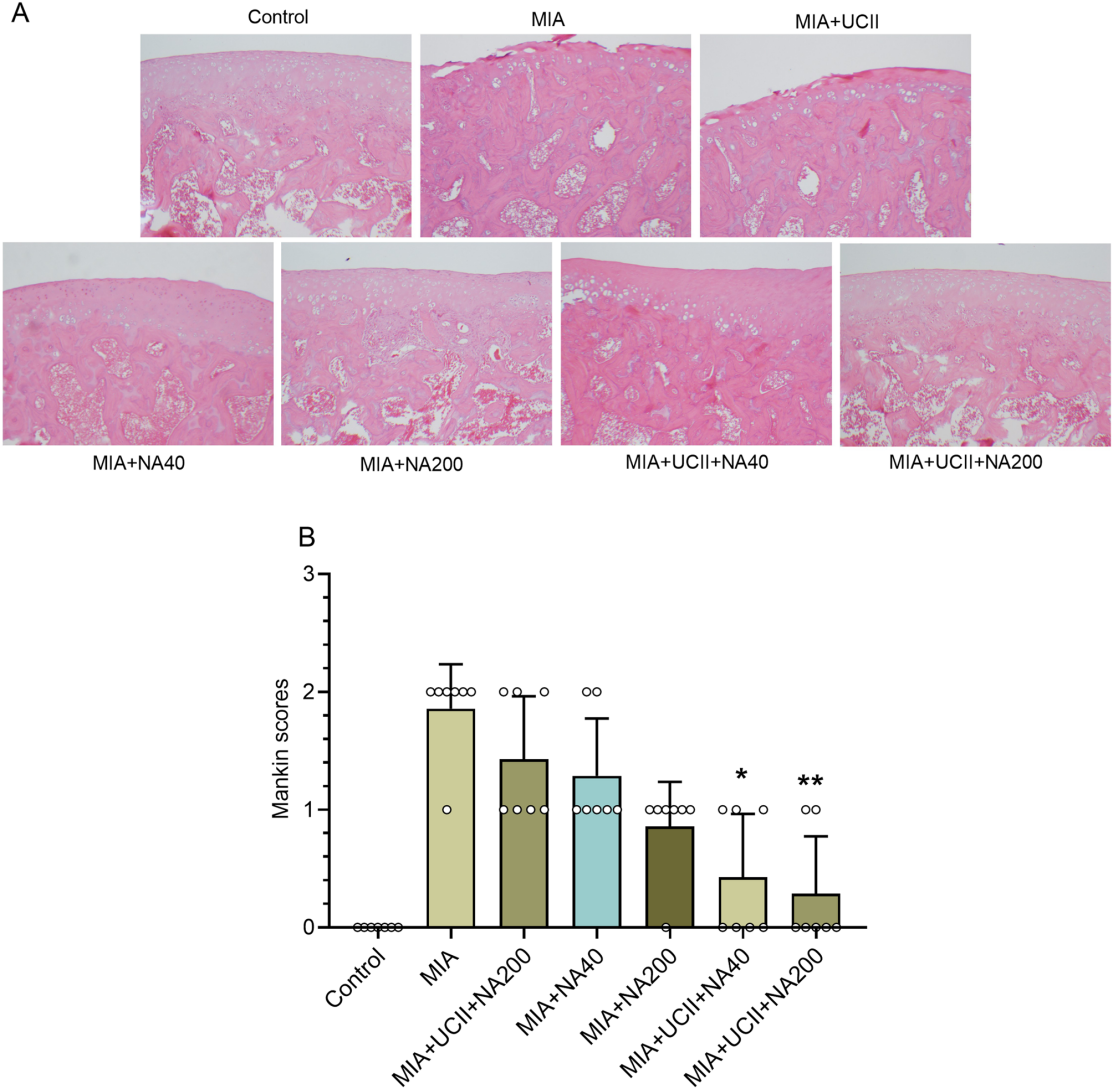
Effects of niacinamide (NA) and undenatured type II collagen (UCII) supplementation on histopathology of the knee joint in monosodium iodoacetate (MIA)-induced osteoarthritis in rats. Representative histopathologic images of hematoxylin–eosin **(A)** obtained at the end of the experiment are shown. Mean values of Mankin scores are demonstrated with ± standard deviations **(B)**. Asterisks above the groups indicate statistical differences (Kruskal–Wallis followed by Mann–Whitney U; * *p* < 0.05; ** *p* < 0.01; compared as MIA group). Control as a non-supplemental group with no osteoarthritis-induced rats, MIA as a non-supplemental group with monosodium iodoacetate (MIA)-induced knee osteoarthritis, MIA + UCII as MIA group gavage-fed a supplemental UCII at 4 mg/kg, MIA + NA40 as MIA group gavage-fed supplemental niacinamide (NA) at 40 mg/kg, MIA + NA200 as MIA group gavage-fed a supplemental NA at 200 mg/kg, MIA + UCII + NA40 as MIA group gavage-fed both supplemental UCII at 4 mg/kg and NA at 40 mg/kg and MIA + UCII + NA200 as MIA group gavage-fed both supplemental UCII at 4 mg/kg and NA at 200 mg/kg. Oral gavage delivery of supplements was applied from d7 to d30.

The joints of the intact rats retained intact superficial and smooth articular cartilage surfaces with the underneath layer of flattened chondrocytes in the tangential zone (Fig. 4). In addition, chondrocytes of the same joints were normally distributed in parallel rows, transitional and radial zones of the articular cartilage. As expected, the rats with OA had irregular surfaces accompanied by loss of cartilage tissue degeneration of the articular cartilage and disappearance of chondrocytes in the tangential, transitional and radial zones of the cartilage. However, supplemental UCII and NA altered the histological changes in rats with OA. The elevated Mankin scores in osteoarthritic rats were decreased with each supplement alone, but further decreases were observed with the combinations of the supplements, particularly with the treatment of UCII + NA200 (*p* < 0.05).

**Figure 5:**
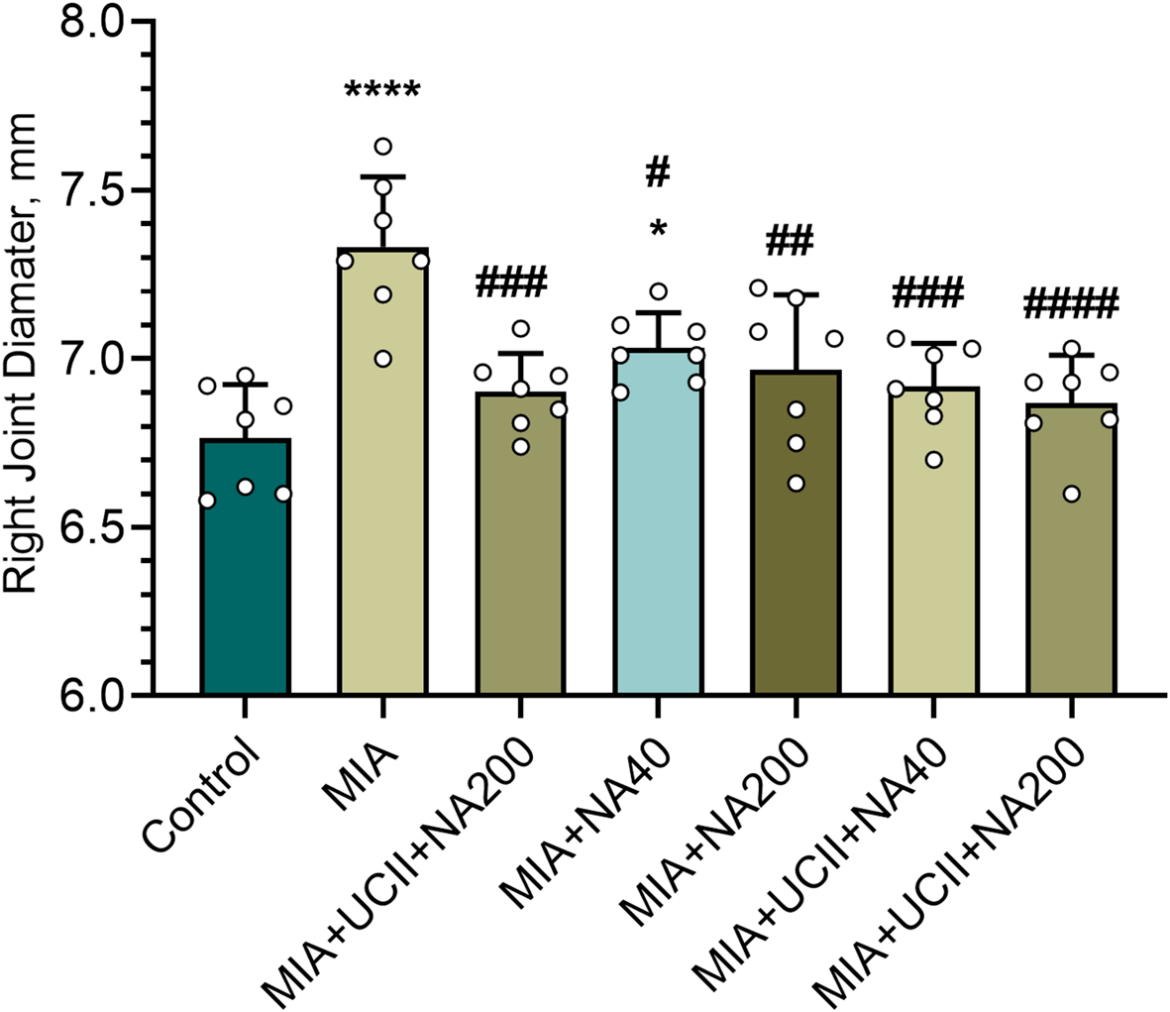
Effects of niacinamide (NA) and undenatured type II collagen (UCII) supplementation on knee joint diameter in monosodium iodoacetate (MIA)-induced osteoarthritis in rats (n = 7). Control as a non-supplemental group with no osteoarthritis-induced rats, MIA as a non-supplemental group with monosodium iodoacetate (MIA)-induced knee osteoarthritis, MIA + UCII as MIA group gavage-fed a supplemental UCII at 4 mg/kg, MIA + NA40 as MIA group gavage-fed supplemental niacinamide (NA) at 40 mg/kg, MIA + NA200 as MIA group gavage-fed a supplemental NA at 200 mg/kg, MIA + UCII + NA40 as MIA group gavage-fed both supplemental UCII at 4 mg/kg and NA at 40 mg/kg and MIA + UCII + NA200 as MIA group gavage-fed both supplemental UCII at 4 mg/kg and NA at 200 mg/kg. Oral gavage delivery of supplements was applied from d7 to d30. The error bars point out the standard deviation of the mean. (ANOVA and Tukey’s post-hoc test were used for comparing the results among different treatment groups. Statistical significance between groups is shown by: *p < 0.05; ****p < 0.0001 compared as Control group and, ^#^*p* < 0.05; ^##^*p* < 0.01; ^###^*p* < 0.001; ^####^*p* < 0.0001 compared as MIA group and (ANOVA and Tukey's post-hoc test; *p* < 0.05).

The right knee joint diameters increased in rats with OA compared with those of control (*p* < 0.05; Fig. 5). Each supplement with a similar extend reduced the knee joint diameter (*p* < 0.05).

**Figure 6:**
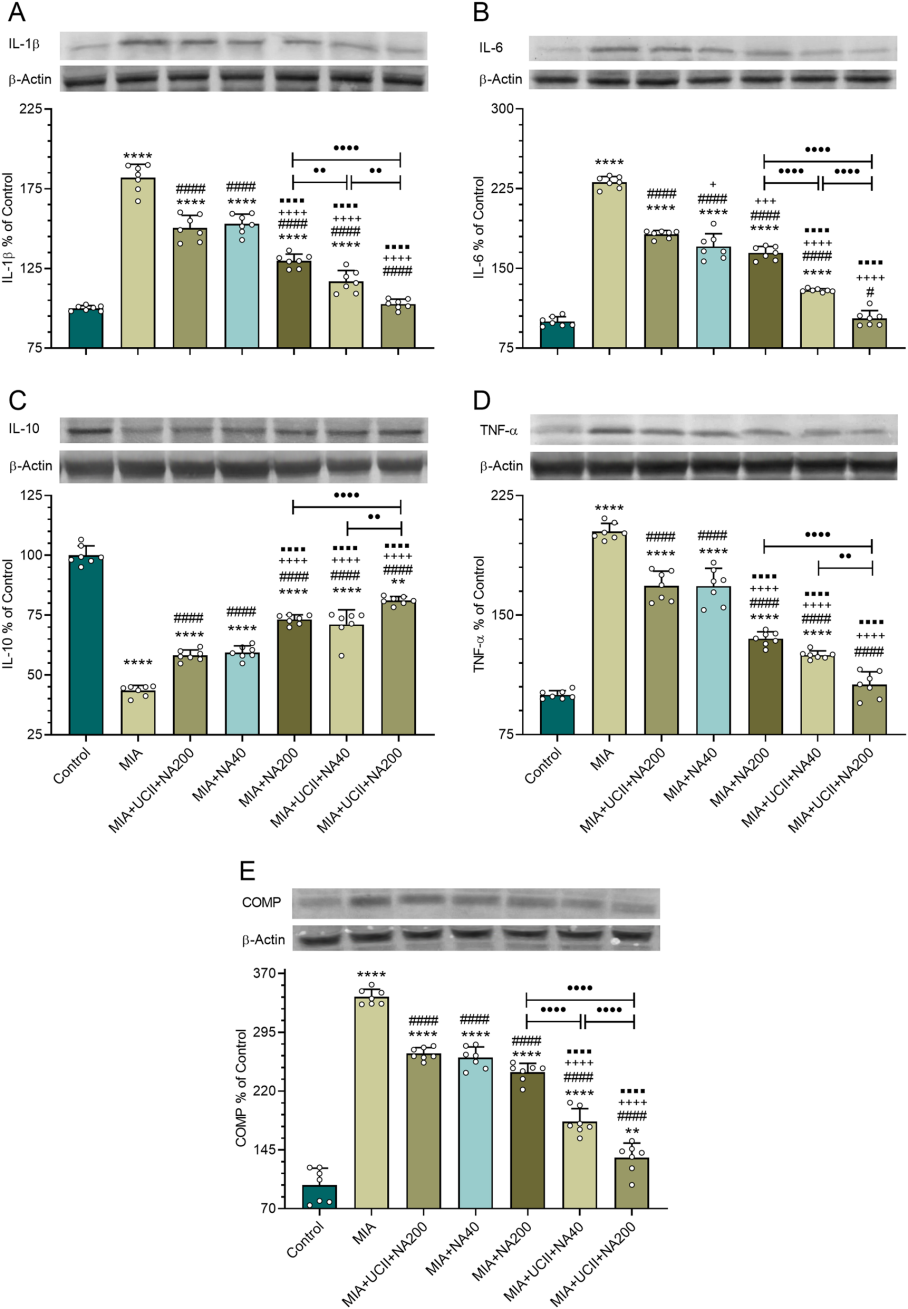
Effects of niacinamide (NA) and undenatured type II collagen (UCII) supplementation on knee joint IL-1β (**A**), IL-6 (**B**), IL-10 (**C**), TNF-α (**D**), and COMP (**E**) levels in monosodium iodoacetate (MIA)-induced osteoarthritis in rats. The densitometric analysis of the relative intensity according to the control group of the western blot bands was performed with β-actin normalization to ensure equal protein loading. Blots were repeated at least three times (n = 3), and a representative blot is shown. Data are expressed as a percent of the control set at 100%. Control as a non-supplemental group with no osteoarthritis-induced rats, MIA as a non-supplemental group with monosodium iodoacetate (MIA)-induced knee osteoarthritis, MIA + UCII as MIA group gavage-fed a supplemental UCII at 4 mg/kg, MIA + NA40 as MIA group gavage-fed supplemental niacinamide (NA) at 40 mg/kg, MIA + NA200 as MIA group gavage-fed a supplemental NA at 200 mg/kg, MIA + UCII + NA40 as MIA group gavage-fed both supplemental UCII at 4 mg/kg and NA at 40 mg/kg and MIA + UCII + NA200 as MIA group gavage-fed both supplemental UCII at 4 mg/kg and NA at 200 mg/kg. Oral gavage delivery of supplements was applied from d7 to d30. The error bars point out the standard deviation of the mean. (ANOVA and Tukey’s post-hoc test were used for comparing the results among different treatment groups. Full-length blots are presented in Supplementary Fig. S2. Statistical significance between groups is shown by: **p < 0.01; ****p < 0.0001 compared as Control group and, ^#^*p* < 0.05; ^####^*p* < 0.0001 compared as MIA group and, ^+^*p* < 0.05; ^+++^*p* < 0.001; ^++++^*p* < 0.0001 compared as MIA + UCII group and, ^⬛⬛⬛⬛^*p* < 0.0001 compared as MIA + NA40 group and, ••*p* < 0.01; ••••*p* < 0.0001 compared as pairwise comparisons between the groups). (ANOVA and Tukey's post-hoc test; *p* < 0.05).

The knee joint IL-1β, IL-6, IL-10, TNF-α, and COMP protein expression levels are reported in Fig. 6. The protein expressions of IL-1β, IL-6, TNF-α, and COMP increased while that of IL-10 decreased (*p* < 0.05) in rats with OA compared with those of control. Each supplement, particularly the combinations with the UCII + NA200 treatment, as being most effective, reversed the responses (*p* < 0.05). The UCII + NA200 supplementation brought the protein expression levels of IL-1β, IL-6, and TNF-α to those of control.

**Figure 7:**
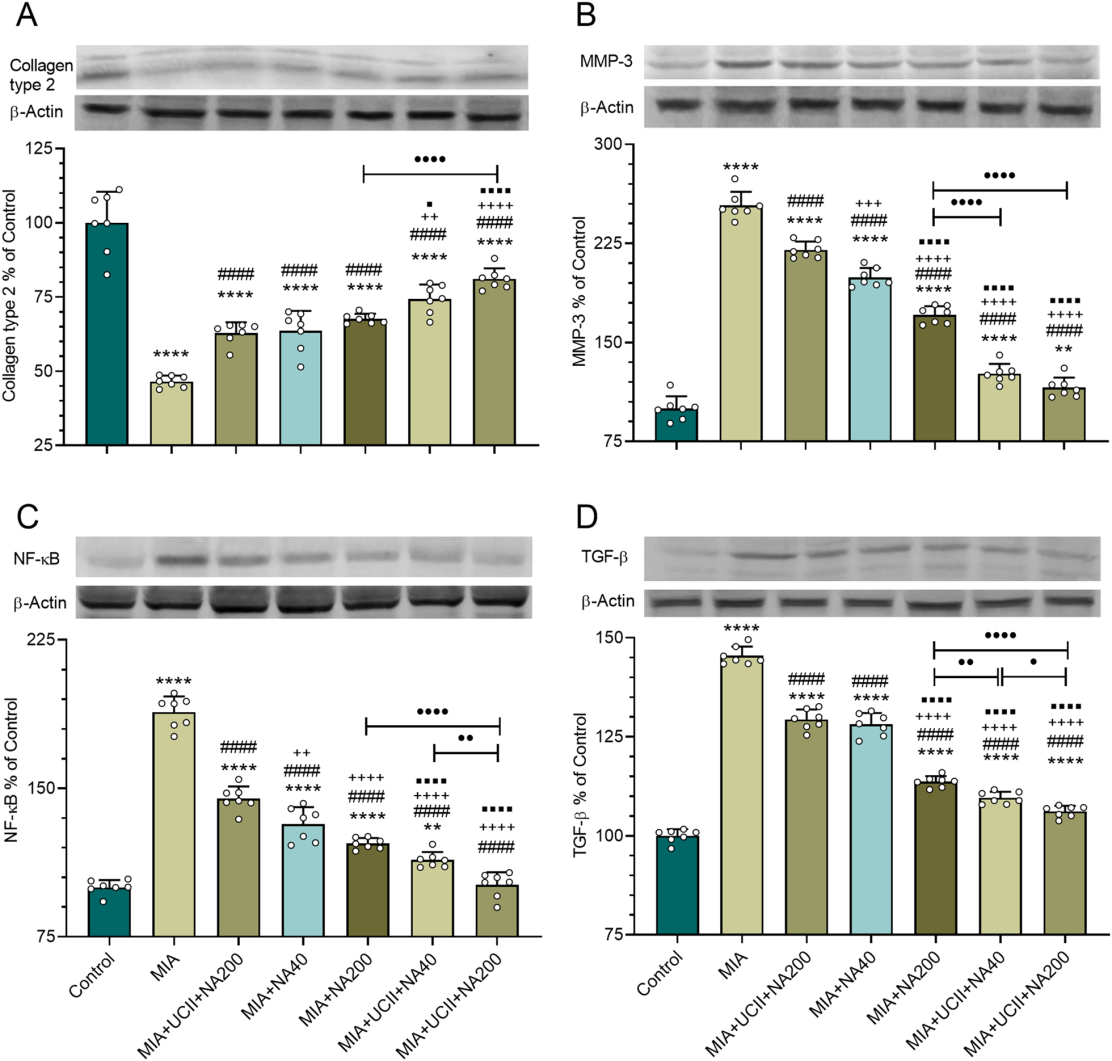
Effects of niacinamide (NA) and undenatured type II collagen (UCII) supplementation on the knee joint collagen type II (**A**), MMP-3 (**B**), NF-κB (**C**), and TGF-β1 (**D**) levels in monosodium iodoacetate (MIA)-induced osteoarthritis in rats. The densitometric analysis of the relative intensity according to the control group of the western blot bands was performed with β-actin normalization to ensure equal protein loading. Blots were repeated at least three times (n = 3), and a representative blot is shown. Data are expressed as a percent of the control set at 100%. Control as a non-supplemental group with no osteoarthritis-induced rats, MIA as a non-supplemental group with monosodium iodoacetate (MIA)-induced knee osteoarthritis, MIA + UCII as MIA group gavage-fed a supplemental UCII at 4 mg/kg, MIA + NA40 as MIA group gavage-fed supplemental niacinamide (NA) at 40 mg/kg, MIA + NA200 as MIA group gavage-fed a supplemental NA at 200 mg/kg, MIA + UCII + NA40 as MIA group gavage-fed both supplemental UCII at 4 mg/kg and NA at 40 mg/kg, and MIA + UCII + NA200 as MIA group gavage-fed both supplemental UCII at 4 mg/kg and NA at 200 mg/kg. Oral gavage delivery of supplements was applied from d7 to d30. The error bars point out the standard deviation of the mean. (ANOVA and Tukey’s post-hoc test were used for comparing the results among different treatment groups. Full-length blots are presented in Supplementary Fig. S3. Statistical significance between groups is shown by: **p < 0.01; ****p < 0.0001 compared as Control group and, ^####^*p* < 0.0001 compared as MIA group and, ^++^*p* < 0.01; ^+++^*p* < 0.001; ^++++^*p* < 0.0001 compared as MIA + UCII group and, ^⬛^*p* < 0.05; ^⬛⬛⬛⬛^*p* < 0.0001 compared as MIA + NA40 group and, •*p* < 0.05; ••*p* < 0.01; ••••*p* < 0.0001 compared as pairwise comparisons between the groups) (ANOVA and Tukey's post-hoc test; *p* < 0.05).

The knee joint MMP-3, NF-κB, and TGB levels increased, whereas collagen type II level decreased in rats with OA (*p* < 0.05; Fig. 7). Each supplement, particularly the combinations with the UCII + NA200 treatment, as being most effective, reversed the responses (*p* < 0.05). The UCII + NA200 supplementation brought the protein expression levels of NF-κB to those of control.

## Discussion

Serum IL‐1β, IL‐6, TNF-α, COMP, and CRP concentrations increased 235%, 438%, 283%,457%, and 595%, respectively in rats with OA compared with those of intact rats, indicating inflammation due to OA Increased production of IL‐1β as a typical proinflammatory cytokine in the damaged joints^30^ was expected in rats with OA As evidenced in the present work, IL‐1β was also demonstrated to lead the secretion of other cytokines such as TNFα, IL-6, and IL-8^31^. The knee joint IL-1β, IL-6, IL-10, TNF-α, and COMP levels were in accord with those of serum. Parallel to the present work results and the common notion, Chandran et al.^32^ also found greater serum concentrations of IL‐1β, IL‐6, and IL‐8 in patients with OA. However, the same authors^20^ detected no changes in TNF-α, COMP, and CRP concentrations. When the rats with OA received the supplementation of UCII and NA each alone but particularly the combination of UCII + NA200, the inflammation was ameliorated. Although there have been no reports of serum inflammation parameters measured in osteoarthritic rats supplemented with collagen in the literature, the present work revealed that rats supplemented with 4 mg UCII/kg body weight as single or combination with niacinamide at either 40 or 200 mg/kg mitigated the inflammation. Niacinamide supplementation for the treatment of OA is scarce in the literature. Osteoarthritic patients treated with niacinamide for 12 weeks lessened inflammation along with decreased severity of OA and improved joint flexibility^8^.

Coherent results to IL‐1β, IL‐6, TNF-α, COMP, and CRP concentrations were also observed at the present work with increasing MDA concentrations but decreasing antioxidant enzyme activities in rats with OA supplementing UCII and NA, as single or as a combination, altered the measured parameters. Increased MDA concentrations can also be used as a sensitive marker for inflammatory damage in arthritis^33^, besides other such specific markers as IL‐1β, IL‐6, TNF-α, COMP, and CRP. Similar to the results of the present work, Jaleel et al.^34^ found elevated serum MDA, IL‐1β, IL‐6, and TNF-α concentration in rats with OA, compared with those of intact rats, and observed that supplementing type III collagen to the rats at 10 mg/kg for two weeks reversed the responses.

The rats with OA bore elevated joint diameters with joint pain behavior measured as decreased the stride lengths, the paw areas, and the paw widths, and increased the Kellgren-Lawrence Mankin scores. The Kellgren-Lawrence score as the measurement of the severity of OA was high, as expected, in rats with OA. However, osteoarthritic rats with supplements, particularly with UCII + NA200, had lower Kellgren-Lawrence scores. Similarly, Bagi et al.^12^ found that oral supplementation of UCII to osteoarthritic rats alleviated articular cartilage's worsening.

Reduced gait patterns in osteoarthritic rats have also been reported^35,36^, with reduced paw areas and paw widths along with decreases in stride length. In addition, changes in gait were observed as a result of increased pain in osteoarthritic mice^37^. Supplementing either UCII or NA, each alone but particularly the combination of UCII + NA200, ameliorated joint pain, especially with the longer treatment duration (d21). Similarly, NA supplementation in patients with OA improved the severity of the OA by 29%, with increasing joint mobility by 4.5 degrees^8^.

Monosodium iodoacetate injection into the joints results in degenerative changes in articular cartilage via matrix degradation and disturbance of chondrocyte metabolism and subsequently chondrocyte death. These events occur mainly due to the inhibition of glyceraldehyde-3-phosphate dehydrogenase activity and thus glycolysis^38^ as well as hydration of the extracellular matrix, and reduced quantity and synthesis of proteoglycans, all leading eventually to cell death^39,40^. As observed in the present study, the sustainability of the cartilage with the death of chondrocytes in osteoarthritic rats was impeded, and disrupted maintenance of the cartilage was restored with each supplement but particularly with the combination of UCII + NA200 through the reduction of joint space narrowing and cartilage destruction. The UCII treatment alone in rats with OA slightly improved cartilage microstructure, degeneration, and surface organization, all of which were parallel to the results of previous works in rats^12^ and mice^41^ with OA. Although its precise mechanism is unknown, niacinamide was speculated to penetrate the cartilage matrix by elevating NAD and NADP levels in synovial fluid^42^. Therefore, nutritional supplementation of NA, as was the case with the current work, would provide energy and nucleic acids through non-oxidative mechanisms (i.e., via the pentose shunt, bypassing the tricyclic acid and glycolytic sequences) that are vital for cartilage repair in the deeper layers of the matrix^43^.

Type II collagen comprises about 90% of the total collagen in hyaline cartilage, also known as articular cartilage damaged in OA^44^. Therefore, decreases in the protein expression of type II collagen are signs of OA, as evidenced in the present work. Progression of OA in the cartilages is related to inflammatory cytokines such as IL‐1β and TNF-α, leading to MMPs (1, 3, 9, and 13) expressions^45^. This was also a case in the present study. Similar to the current work results, Davidson et al.^46^ found increased protein expression of TGF-β1 in synovial cells of osteoarthritic mice. Similarly, mRNA expression levels of Tgfb1 genes were reported increased in the knee cartilage of rats with MIA-induced OA^47^. The supplementation of UCII and NA, each alone but particularly the combination of UCII + NA200, increased the synthesis of type II collagen and reduced the inflammation parameters in the knee joint.

Apparently, both UCII and NA, each alone but mainly as a combination, regenerated the knee cartilage, mitigating the inflammation and helping normal functions of joints and tendons. The effects of the UCII could be due to its rich glycine contents and proline, which are required for the normal function of joints and tendons^48^. Extreme decreases of collagen synthesis in osteoarthritis have been shown due to severe glycine deficiency, and increased glycine concentrations in vitro have been indicated to advance collagen synthesis and consequently cartilage regeneration^49^.

The mechanism of NA in the scenario of OA at the present work and in the literature has not been explored. The current work provided evidence that NA acts similar to that of UCII in most parameters measured in alleviating the symptoms of knee OA Niacinamide has been speculated to inhibit the synthesis and/or activity of IL-1^14^. In addition, NA has been considered a part of antioxidant defense mechanisms^4–6^ and is evidenced in the present work. Niacinamide treatment has also been reported to be beneficial in treating osteoarthritis with better joint flexibility and decreased inflammation^8^. However, more work has to be conducted in clearing up the detailed mechanism of NA in arthritis.

In general, UCII alone is better than NA alone in improving the paw width, stride length, and numerical values of inflammatory serum parameters. However, NA alone, particularly with greater doses, was better than that of U-II alone in knee joint inflammation parameter levels as well as Collagen Type II, MMP-3, NF-κB, and TGB levels. The effects of UCII and NA were most probably linked to the suppression of the production of proinflammatory cytokines and mediators such as IL‐1β, IL‐6, TNF-α, COMP, and CRP. Rats with MIA-induced OA were reported to have greater NF-κB1 gene expression levels, compared with those of healthy rats, in articular cartilages, subchondral bone, and synovial membrane of the rat knee joint^50,51^. Therefore, inhibition of NF-κB1 expressions in cartilage can counteract chondrocytes damage through increased synthesis of proinflammatory cytokines^52^. The supplements were also involved in type II collagen synthesis, helping to alleviate the symptoms of OA. It is still a question of how much of each effect (increased type II collagen synthesis or a reduction in inflammation parameters) contributed more to alleviating OA symptoms and which supplement is more effective. Therefore, further work is required to explore the detailed molecular mechanisms of action of UCII and NA.

In vitro results (Supplementary Tables S1, S2 and Supplementary Fig. S1) conducted for this work were in accord with the results of the present experiment. Reduction of inflammatory markers including IG6, COX2, TNF-α, and NF-κB was observed when a combination of collagen and niacinamide was used in THP-1 monocyte cells differentiated into macrophages and treated with various extracts as control (0.2% DMSO–0.2%, v/v), UCII (50 ug/mL), Niacinamide (50 ug/mL), UCII + Niacinamide (50 ug/mL + 50 ug/mL), and Rosiglitazone (5 mM) (Supplementary Table S2).

Supplementing a combination of UCII at 4 mg/kg and niacinamide at 200 mg/kg for three weeks is a promising dietary strategy for reducing pain, minimizing cartilage damage improving functional status in knee OA of rat models. The results can also be applied to humans suffering from knee OA, being an alternative to conventional drug treatments. The mechanism by which how the supplements improve functional status in knee OA of rat models needs to be elucidated through clinical investigations.

## Supplementary Information

Supplementary Information.

## Data Availability

The datasets used and analyzed during the current study are available from the corresponding author on reasonable request.
