# Supplementary material for: Niacinamide and undenatured type II collagen modulates the inflammatory response in rats with monoiodoacetate-induced osteoarthritis
Source: Sci Rep. 2021 Jul 19;11:14724. doi: 10.1038/s41598-021-94142-3 (PMC8289820; doi:10.1038/s41598-021-94142-3)
Supplement: Supplementary file 1 — Supplementary Information. [file 41598_2021_94142_MOESM1_ESM.docx]

**SUPPLEMENTARY FILES**

Niacinamide and undenatured type II collagen modulates the inflammatory response in rats with monoiodoacetate-induced osteoarthritis

Kazim Sahin ^1,^*, Osman Kucuk ^2^, Cemal Orhan^1^, Mehmet Tuzcu ^3^, Ali Said Durmus^4^, Ibrahim Hanifi Ozercan^5^ Nurhan Sahin^1^ and Vijaya Juturu^6^

^1^ Department of Animal Nutrition, Faculty of Veterinary Medicine, Firat University, Elazig, Turkey; nsahinkm@yahoo.com (K.S.); corhan@firat.edu.tr; (C.O); nsahin@firat.edu.tr (NS).

^2^ Department of Animal Nutrition, Faculty of Veterinary Medicine, Erciyes University, Kayseri, Turkey; osmankucukwy@yahoo.com (O.K).

^3^ Department of Biology, Faculty of Science, Firat University, Elazig, Turkey; mtuzcu@firat.edu.tr (M.T.).

^4^ Department of Surgery, Faculty of Veterinary Medicine, Firat University, Elazig, Turkey; asdurmus@firat.edu.tr (A.S.D.).

^5^ Department of Pathology, Faculty of Medicine, Firat University, Elazig, Turkey; ozercanih@yahoo.com (I.H.O.).

^6^ Lonza Inc., Consumer Health & Nutrition, Morristown, New Jersey, U.S.A.; vijaya.juturu@lonza.com

* Correspondence: Email: nsahinkm@yahoo.com, ksahin@firat.edu.tr; Phone: +90-532-7473506, Phone: +904242370000/3938.

*Correspondence to: Kazim Sahin, DVM, Ph.D., Member of The Turkish Academy of Sciences Professor of Nutrition Veterinary Faculty Firat University 23119 Elazig, Turkey Phone:+90-532-7473506 Phone:+904242370000/3938

Fax:+904242388173, Email: nsahinkm@yahoo.com, ksahin@firat.edu.tr

**Supplementary Table S1.** *In vitro* data for cytotoxicity

| **Dose, ug/mL** | **Niacinamide** | **UCII** | **Stauro.** |
| --- | --- | --- | --- |
| **100** | 287.5±0.9 | 297.0±8.7 | 90.0±12.0 |
| **50** | 288.9±15.0 | 254.6±25.7 | 159.3±0.6 |
| **25** | 310.1±2.8 | 282.9±36.6 | 196.3±15.1 |
| **12.5** | 290.4±5.0 | 310.1±8.9 | 217.8±8.8 |
| **6.25** | 315.7±9.8 | 313.0±7.7 | 262.8±14.8 |
| **3.125** | 319.2±7.5 | 283.6±25.9 | 305.0±0.3 |
| **1.5625** | 303.7±7.4 | 285.4±7.5 | 314.2±17.5 |
| **0** | 314.4±11.3 | 315.0±17.2 | 292.0±8.9 |
| Data are presented as mean and standard deviation.  UCII: undenatured type II collagen | | | |

**Supplementary Figure S1.** *In vitro* data for cell viability (%).

**Supplementary Table S2.** *In vitro* data for protein expressions

| Groups | IL-6 | COX2 | TNF-α | NF-κB | IL-1RA |
| --- | --- | --- | --- | --- | --- |
| Control | 1.0130± 0.118 | 1.0060±0.079 | 1.0140±0.125 | 1.0020±0.045 | 1.0253±0.165 |
| UCII | 1.1724±0.117 | 1.6860±0.467 | 1.1344±0.045 | 0.9760±0.048 | 1.2450±0.159 |
| NIA | 1.2708±0.121 | 1.0457±0.059 | 1.2445±0.121 | 1.1270±0.052 | 1.1043±0.062 |
| UCII+NIA | 1.0697±0.067 | 0.8757±0.049 | 1.0125±0.058 | 1.0563±0.063 | 0.9967±0.072 |
| Rosiglitazone | 1.2525±0.064 | 1.3950±0.112 | 0.8530±0.147 | 0.8253±0.083 | 1.0860±0.137 |
| IL-6: Interleukin 6; COX2: Cyclooxygenase-2; TNFα: Tumor necrosis factor alpha; NF-κB: Nuclear Factor kappa B; IL-1RA: Interleukin-1 Receptor Antagonist.  Control: 0.2% DMSO; UCII: undenatured type II collagen, 50 µg/mL; NIA: Niacinamide, 50 µg/mL; Rosiglitazone, 5mM.  Data are presented as mean and standard error. | | | | | |

IL-1β


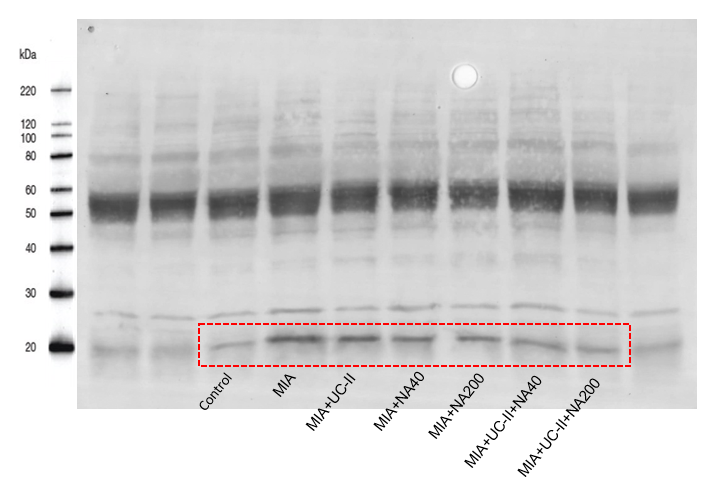


β-actin


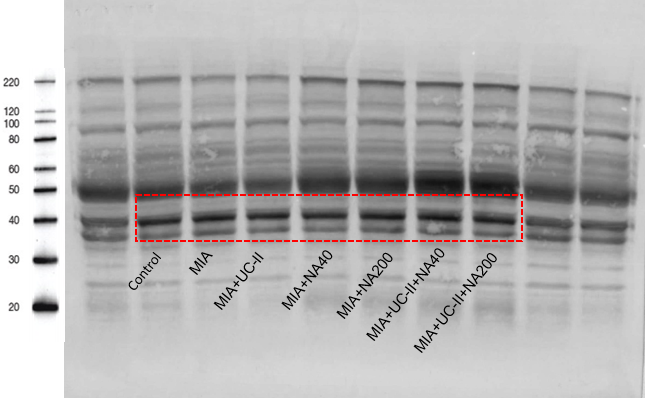


IL-6


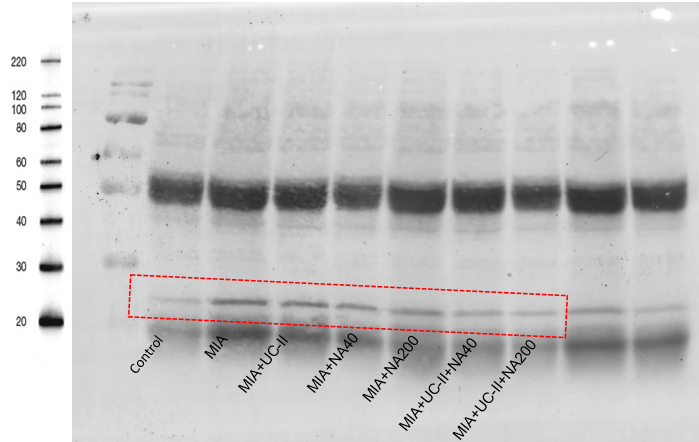


β-actin


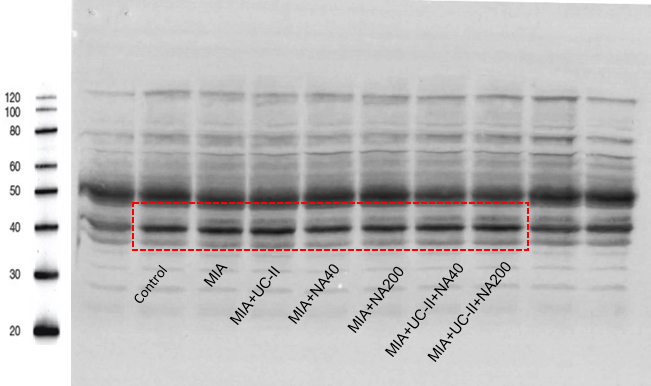


IL-10


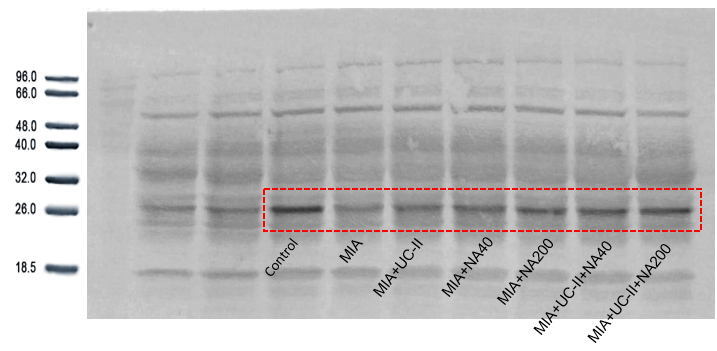


β-actin


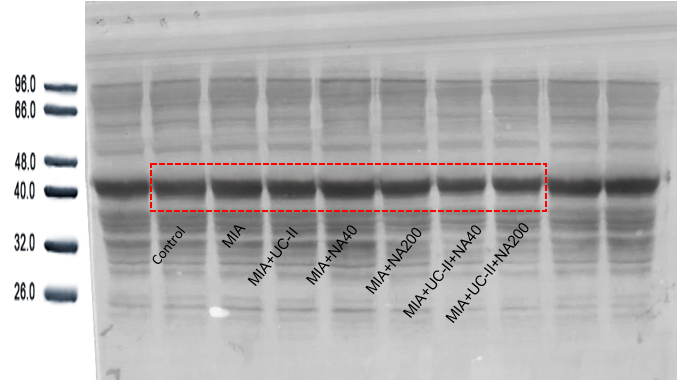


TNF-α


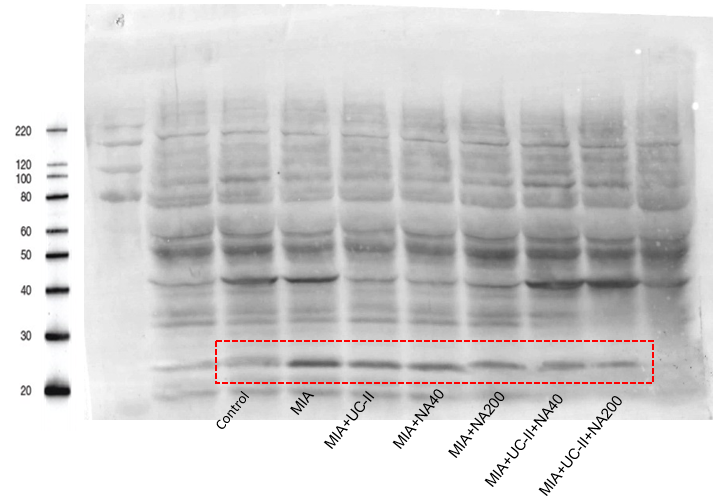


β-actin


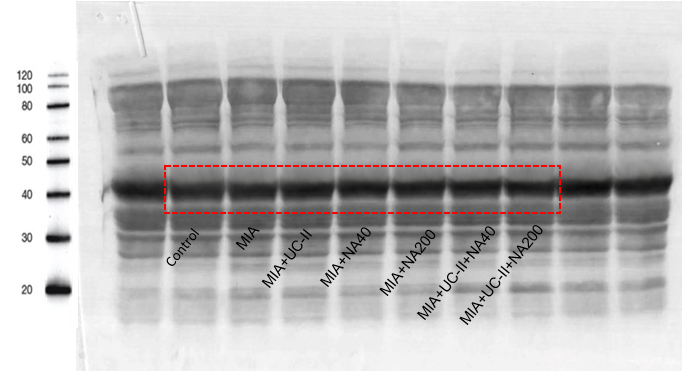


COMP


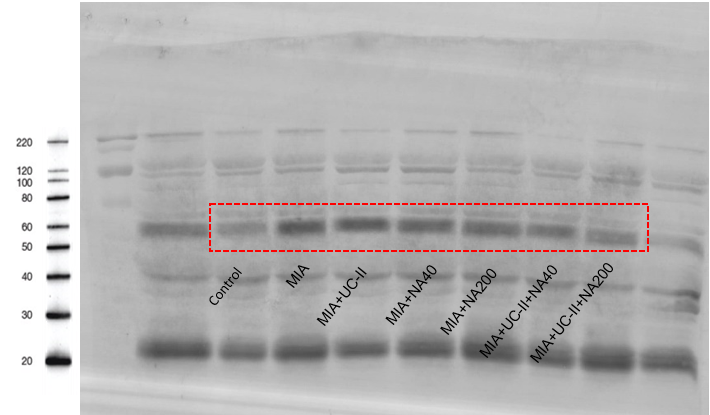


β-actin


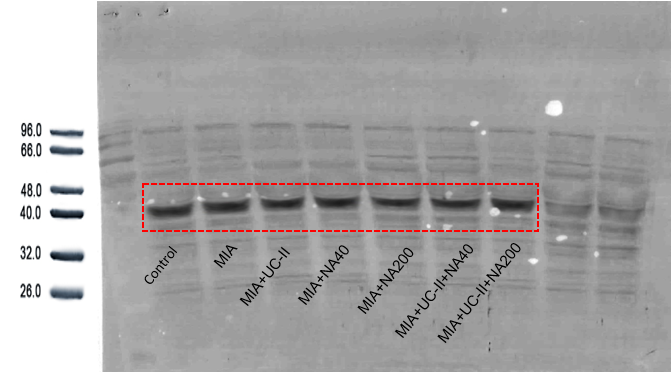


**Supplementary Figure S2.** Raw western blot image of joint tissue levels of interleukin **(**IL)-1β, IL-6, IL-10, tumor necrosis factor (TNF)-α, and Cartilage oligomeric matrix protein (COMP) in rats (correspond to Fig 7 in the manuscript). Results shown in Figure 6 are delineated by red dotted rectangles. MW (in kDa) are indicated.

**C**ollagen type 2


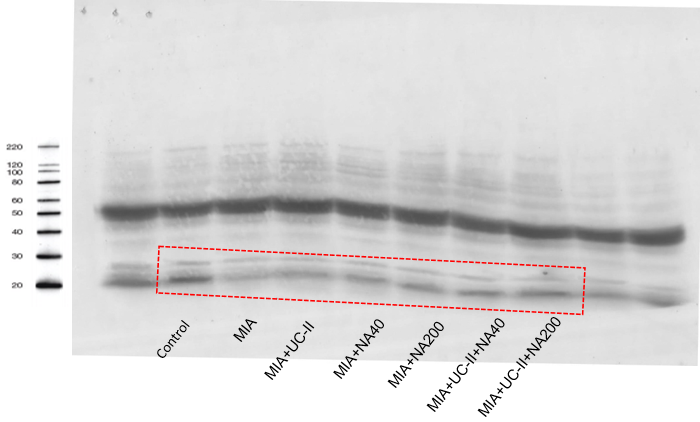


β-actin


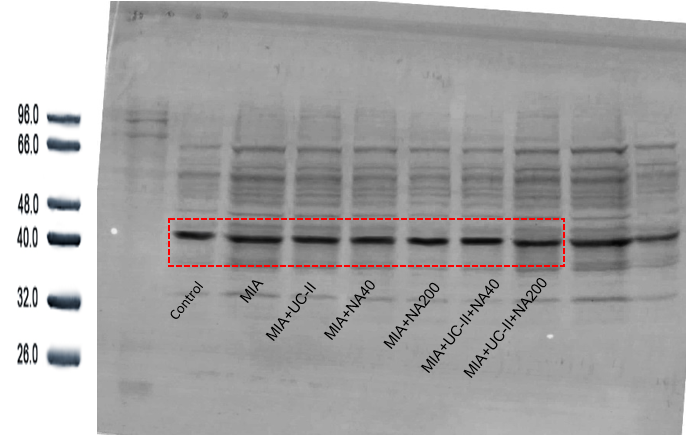


MMP3


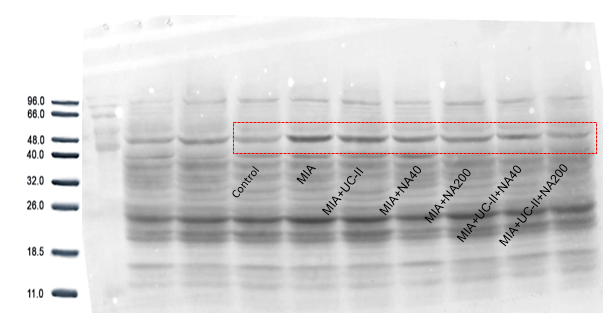


β-actin


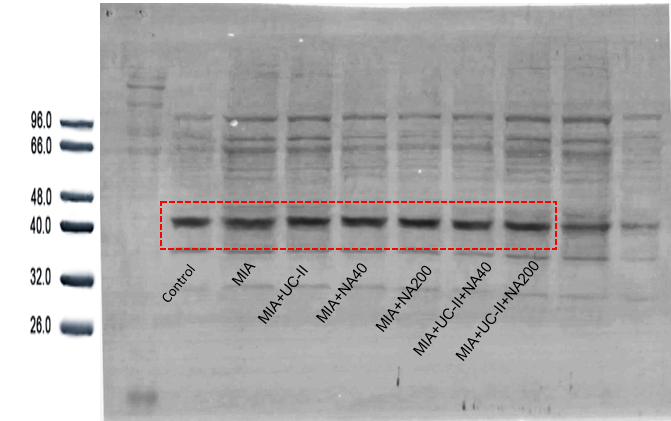


NF-κβ


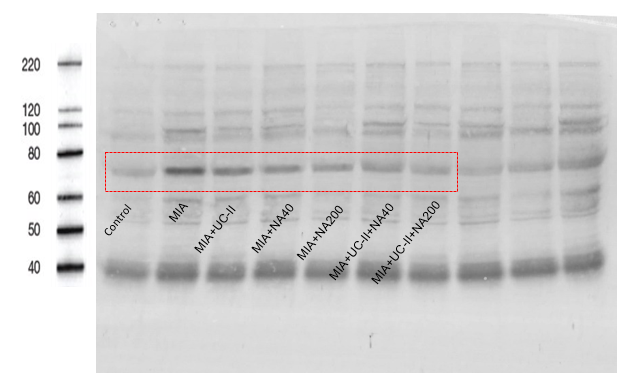


β-actin


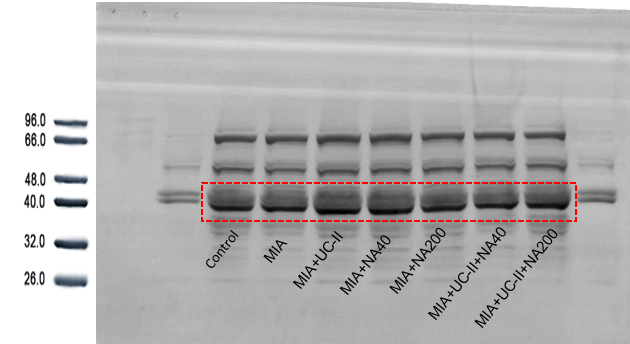


TGF-β


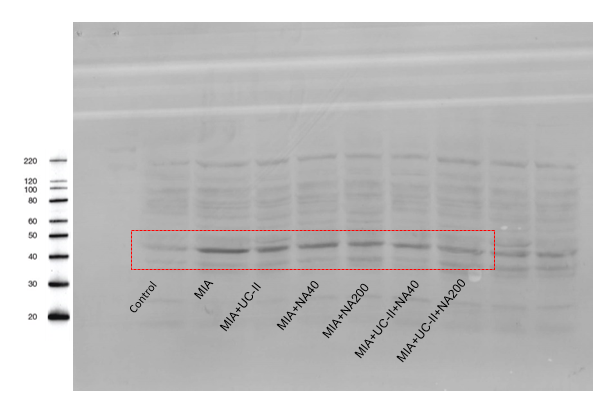


β-actin


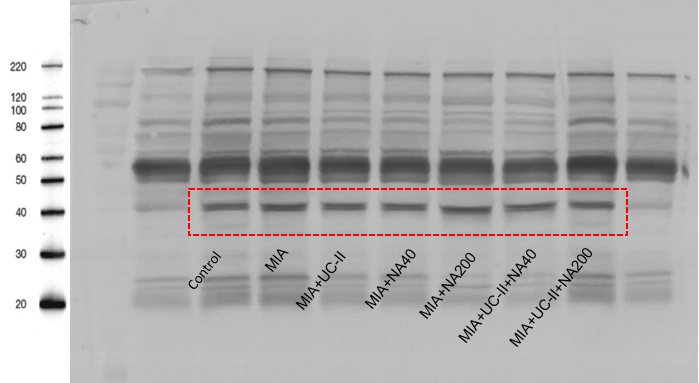


**Supplementary Figure S3.** Raw western blot image of joint tissue levels of **c**ollagen type 2, matrix metalloproteinase-3 (MMP-3), nuclear factor kappa (NF-κβ), and transforming growth factor β (TGF-β) (correspond to Fig 7 in the manuscript). Results shown in Figure 7 are delineated by red dotted rectangles. MW (in kDa) are indicated.
